# Insight into the Molecular Interaction of Cloxyquin (5-chloro-8-hydroxyquinoline) with Bovine Serum Albumin: Biophysical Analysis and Computational Simulation

**DOI:** 10.3390/ijms21010249

**Published:** 2019-12-30

**Authors:** Kamonrat Phopin, Waralee Ruankham, Supaluk Prachayasittikul, Virapong Prachayasittikul, Tanawut Tantimongcolwat

**Affiliations:** 1 Center for Research and Innovation, Faculty of Medical Technology, Mahidol University, Bangkok 10700, Thailand; 2 Department of Clinical Microbiology and Applied Technology, Faculty of Medical Technology, Mahidol University, Bangkok 10700, Thailand; 3 Center of Data Mining and Biomedical Informatics, Faculty of Medical Technology, Mahidol University, Bangkok 10700, Thailand

**Keywords:** cloxyquin, 8-hydroxyquinoline derivatives, serum albumin, fluorescence quenching, biophysical analysis, molecular docking

## Abstract

Cloxyquin is a potential therapeutic compound possessing various bioactivities, especially antibacterial, antifungal, cardioprotective, and pain relief activities. Herein, the interaction mechanism between cloxyquin and bovine serum albumin (BSA) has been elucidated in order to fulfill its pharmacokinetic and pharmacodynamic gaps essential for further development as a therapeutic drug. Multi-spectroscopic and biophysical model analysis suggested that cloxyquin interacts with BSA via a static process by ground-state complex formation. Its binding behavior emerged as a biphasic fashion with a moderate binding constant at the level of 10^4^ M^−1^. Thermodynamic analysis and molecular docking simulation concurrently revealed that hydrophobic interaction is a major driving force for BSA–cloxyquin complexation. Binding of cloxyquin tends to slightly enlarge the monomeric size of BSA without a significant increase of aggregate fraction. Cloxyquin preferentially binds into the fatty acid binding site 5 (FA5) of the BSA via hydrophobic interaction amongst its quinoline scaffold and Phe550, Leu531, and Leu574 residues of BSA. The quinoline ring and hydroxyl moiety of cloxyquin also form the π–π interaction and the hydrogen bond with Phe506. Our data indicate a potential function of serum albumin as a carrier of cloxyquin in blood circulation.

## 1. Introduction

**Figure 1:**
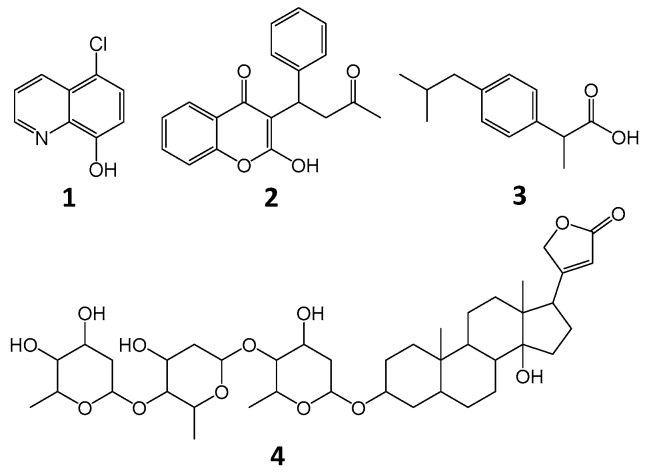
Chemical structures of cloxyquin (**1**), warfarin (**2**), ibuprofen (**3**), and digitoxin (**4**).

Cloxyquin (5-chloro-8-hydroxyquinoline; Figure 1 is a chlorinated derivative of 8-hydroxyquinoline possessing diverse bioactivities and therapeutic potentials. Cloxyquin has been reported to exhibit high bactericidal effect against methicillin-resistant *Staphylococcus aureus* isolated from clinical isolates [1]. It exerts strong antibacterial activities toward *Listeria monocytogenes* (minimum inhibition concentration; MIC = 5.57 µM) and *Plesiomonas shigelloides* (MIC = 11.14 µM) [2]. The antibacterial activity of cloxyquin also extends to *Mycobacterium tuberculosis*, including the multidrug resistant strains, with the MICs in a range of 0.125–0.25 µg/mL [3]. Additionally, cloxyquin also reveals its bioactivities toward eukaryotic systems. Its anti-cryptosporidium [4], antifungal, and antiprotozoal activities [3,5] have been reported. Cloxyquin demonstrates the cardioprotective activity by uncoupling mitochondria and inducing autophagy [6]. Furthermore, cloxyquin-mediated potassium channel activation has been reported. It selectively targets TRESK (K_2P_ channel family) and substantially activates the background potassium current, suggested for further development to be a promising therapeutic ingredient for migraine and other types of nociceptive pain [7,8]. Recently, the neuroprotective effect of cloxyquin derivative (nitroxoline) has been reported in H_2_O_2_-induced human neuronal cells [9]. A polymorph of cloxyquin has been developed to improve its water solubility and dissociation rate [10]. The gelation form of cloxyquin has been established for better drug delivery and pharmaceutical formulation [11]. Currently, information about pharmacokinetic behavior and bioavailability of cloxyquin remains inadequate even if there are several suggestions on its therapeutic roles.

Interaction with serum protein is a key factor influencing the pharmacokinetics and pharmacodynamics of drugs. Serum albumin (SA) is the most abundant protein in circulatory system playing important roles in transportation, distribution, and excretion of both exogenous and endogenous compounds, especially therapeutic drugs [12,13]. Bovine serum albumin (BSA) is traditionally employed as a representative model for studying the binding of drugs with serum proteins due to similarity of its structure to human serum albumin (HSA), ease of availability, and low cost. BSA comprises 583 amino acids forming into three helical domains (I, II, and III domains) as a heart-shaped globular structure. Each domain can be categorized into two subdomains, namely A and B [14]. There are two tryptophan residues, Trp134 and Trp213 located in the subdomains IA and IIA of BSA, respectively [15], in which their intrinsic fluorescence is sensitive to microenvironmental changes [16] and widely employed as an inherent probe for investigation of BSA-drug interaction. There are three major binding pockets of drug that have been identified on the BSA structure and assigned as sites I, II, and III. The site I and site II locate in hydrophobic pockets of subdomains IIA and IIIA, respectively. They preferentially bind with aromatic and heterocyclic compounds [17]. The site III localizes in subdomain IB serving as a major binding site for various compounds, such as derivatives of sulfonamide, hemin, and bilirubin. In addition, seven fatty acid binding pockets have been recognized in subdomains IB, IIIA, IIIB, and subdomain interfaces [17,18]. Elucidating the interaction of drugs with serum albumin provides a better understanding of its actions. Previous study reported that 8-hydroxyquinoline tends to have multiple binding sites on HSA. However, 8-hydroxyquinoline favors to be docked into a pocket at an interface between domains I and II of HSA by networking with Lys106, Gln29, Tyr148, Tyr150, and Cys246 [19]. The nitro [20] and the amino [21] derivatives of 8-hydroxyquinoline prefer to form complex with serum albumin by a moderate affinity at about 10^5^ M^−1^ binding constant. Furthermore, derivative of cloxyquin bearing iodo group at the 7^th^ position (5-chloro-7-iodo-8-hydroxyquinoline or clioquinol) exhibits extreme affinity toward BSA with a binding constant of 10^8^ M^−1^ [22]. To the best of our knowledge, the information about interaction of cloxyquin itself with serum albumin remains unexplored. Therefore, this work aimed to gain precious knowledge of pharmacodynamics and pharmacokinetics of cloxyquin and bring a step closer to its applications. The interaction mechanism of cloxyquin with BSA was extensively investigated using diverse spectroscopic methods (UV-Vis absorption, fluorescence, and CD spectroscopies) in accordance with biophysical model analysis (e.g., Stern–Volmer’s, Hill’s, and thermodynamic equations). In addition, the structural information of cloxyquin-BSA interaction was explored by molecular docking simulation.

## 2. Results and Discussion

### 2.1. Cloxyquin-Induced Fluorescence Quenching of BSA

**Figure 2:**
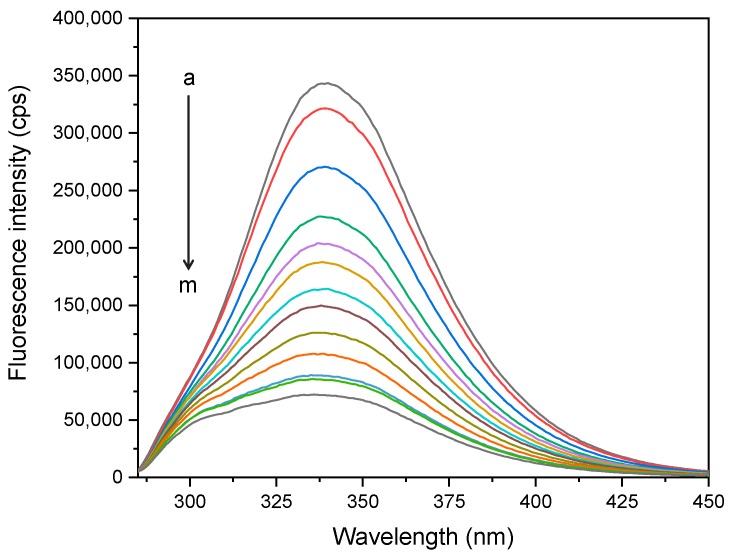
Fluorescence spectra of BSA (4 µM) in the presence of cloxyquin (a–m: 0, 1, 5, 10, 15, 20, 25, 30, 40, 50, 60, 70, and 80 µM) upon excitation with 280 nm wavelength at 310 K.

BSA possesses the intrinsic fluorescence property due to the fluorescence emission of its phenylalanine (Phe), tyrosine (Tyr), and tryptophan (Trp) residues. Tryptophan is a dominant intrinsic fluorophore and very sensitive to local environmental changes as a result of conformational transition, subunit association, complex formation, and denaturation [23]. Fluorescence quenching study provides precious information about the interaction mechanism between protein and ligand [24]. Herein, fluorescence spectra of BSA (4 µM) upon exposure to various concentrations of cloxyquin (0–80 µM) were measured and shown in Figure 2. In the absence of cloxyquin, BSA revealed a typical emission spectrum with a maximum peak at 340 nm when excited at 280 nm wavelength. The fluorescence intensity of BSA was dramatically declined with the increased concentrations of cloxyquin. The peak shift phenomenon was not observed. Nevertheless, a shoulder peak likely appeared at the left edge of the fluorescence spectrum. This observation may suggest the formation of ground-state complex between BSA and cloxyquin [22].

To elucidate the interaction mechanism between BSA and cloxyquin, the Stern–Volmer equation (Equation (1)) was applied to the emission intensity at 340 nm of BSA spectra after exposure to various concentrations of cloxyquin. The plots of *F*_0_/*F* versus ligand concentration ([Q]) fitted with the Stern–Volmer equation typically help to distinguish between dynamic and static interactions between protein and its ligand. The static mechanism means to the ground-state complex formation, and the dynamic mechanism refers to the collisional encountering process. The linear trend of a Stern–Volmer plot suggests that the interaction is driven by single mechanism either static or dynamic process. If the dynamic process mainly involved in the interaction, the Stern–Volmer constant ($K_{sv}$) would be increased as increasing the temperature of interaction system. The rising temperature enhances diffusion and collision of protein and its ligand, which later promotes the dynamic process as indicated by the increased slope of the Stern–Volmer plot. In contrary, the rising temperature destabilizes the protein-ligand complex and substantially reduces the $K_{sv}$ in the static quenching process. In some circumstances, the interaction may not be urged by only one mechanism, but both static and dynamic processes can be simultaneously involved. This phenomenon can be identified by the upward deviation from the linear curve of the Stern–Volmer plot.

(1) $$\frac{F_{0}}{F}=1+K_{sv}\left[Q\right]=1+k_{q}\tau_{0}\left[Q\right]$$

$F_{0}$ and F are the steady-state fluorescence intensities in the absence and the presence of quencher (cloxyquin), respectively. [Q] stands for the concentration of quencher. $K_{sv}$ and $k_{q}$ are the Stern–Volmer constant and the biomolecular quenching rate constant, respectively. $\tau_{0}$ is the fluorescence lifetime of the fluorophore (BSA) in the absence of quencher, generally assigned to be 1 × 10^−8^ s for biopolymers [24].

**Figure 3:**
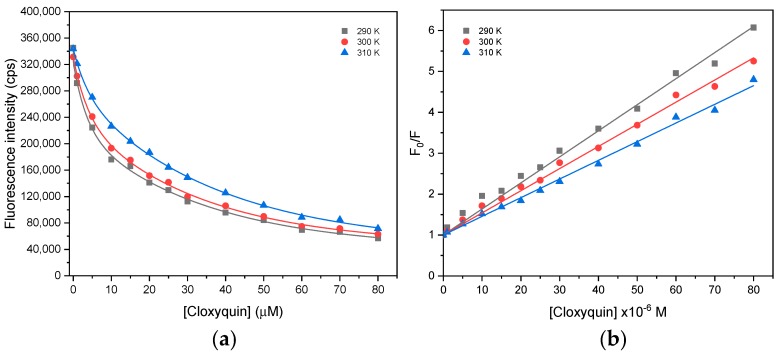
(**a**) The fluorescence quenching and (**b**) the Stern–Volmer plots of bovine serum albumin (BSA) in the presence of cloxyquin at 290, 300, and 310 K. [Cloxyquin] = 0–80 µM; [BSA] = 4 µM; λ_ex_ = 280 nm; λ_em_ = 340 nm.

In this study, the fluorescence quenching was investigated at three difference temperatures (290, 300, and 310 K) by measuring the fluorescence intensity at 340 nm with an excitation wavelength of 280 nm. The fluorescence of BSA was dramatically reduced when exposed to cloxyquin in a range of 1–20 µM and almost saturated at above 50 µM of cloxyquin (Figure 3a). The quenching effect was inversely proportional to the temperature, in which the rising temperature of interaction resulted in the attenuation of fluorescence quenching effect. The plots of $F_{0}/F$ versus cloxyquin concentrations were perfectly fitted with a linear model of the Stern–Volmer equation (*r*^2^ > 0.99; Figure 3b), suggesting a single mechanism involved in the BSA–cloxyquin interaction. The $K_{sv}$ values were decreased as the temperatures increased (Table 1), indicating the primary involvement of static quenching process. Moreover, the biomolecular quenching rate constants ($k_{q}$) were found at a level of 10^12^ M^−1^s^−1^, which were 100 times higher than the maximum scattering collisional quenching constant of various quenchers (2 × 10^10^ M^−1^s^−1^) [25]. Hence, the findings suggested that cloxyquin interacts with BSA via static process by the ground-state complex formation.

To further explore the mechanism of interaction, the fluorescence quenching of BSA as a result of cloxyquin exposure was analyzed by means of the modified Stern–Volmer equation as Equation (2) [26].

(2) $$\frac{F_{0}}{F_{0}-F}=\frac{1}{f_{a}K_{a}}\cdot \frac{1}{\left[Q\right]}+\frac{1}{f_{a}}\;$$

**Figure 4:**
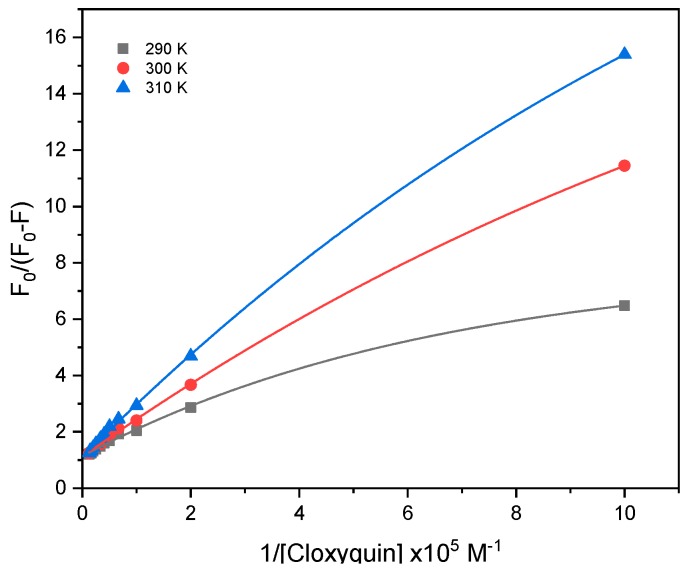
The modified Stern–Volmer plots of BSA after exposed to various concentrations of cloxyquin at 290, 300, and 310 K. [Cloxyquin] = 0–80 µM; [BSA] = 4 µM; λ_ex_ = 280 nm; λ_em_ = 340 nm.

$K_{a}$ is the effective quenching constant for the accessible fluorophores, analogous to the association constant for the quencher-acceptor system. $f_{a}$ is the fraction of accessible fluorescence. $F_{0}$, F, and [Q] serve as the same denotations as the aforementioned in Equation (1). The $F_{0}$/($F_{0}-F)$ versus 1/[Q] plots generated the slight concave downward curves, suggested to be the biphasic binding behavior between cloxyquin and BSA (Figure 4). Accordingly, the $K_{a}$ values were separately determined at low (1–25 µM) and high (15–80 µM) levels of cloxyquin concentrations (Figure S1). At low level of cloxyquin, the $K_{a}$ values were found to be 2–4 times higher than that observed at high level (Table 2). The $f_{a}$ values were close to 0.7 and 1 at low and high concentrations of cloxyquin, respectively. Increasing the temperature of interaction diminished the $K_{a}$ values, but not significantly affected the $f_{a}$ parameters. These findings indicate that cloxyquin binds to BSA with biphasic behavior of the static quenching process and fully occupies the accessible binding sites on BSA at its high concentrations.

### 2.2. BSA and Cloxyquin Absorption Spectra

**Figure 5:**
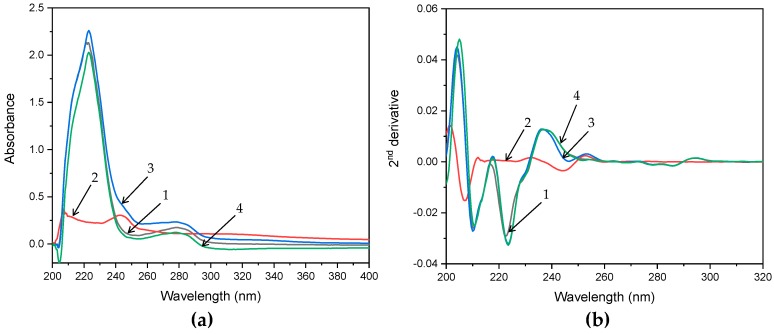
(**a**) Absorption spectra and (**b**) its second order derivatives of BSA (1), cloxyquin (2), BSA–cloxyquin (3), and BSA–cloxyquin subtracted by cloxquin (4). [BSA] = 4 µM, [cloxyquin] = 5 µM, at 310 K, pH 7.4.

To validate the contribution of static quenching process, UV-Vis spectra of BSA with and without cloxyquin at 310 K were analyzed. Static quenching is a complex formation process between ligand and BSA at the ground state level, which typically alters the absorption spectrum of BSA. On the contrary, the process of dynamic quenching only influences the excited state of the fluorophores, so generally there is no absorption spectra change of the BSA. As shown in Figure 5, absorption spectra and its second order derivative transformation revealed that BSA exhibited two distinct absorption peaks at about 222 and 278 nm in the absence of cloxyquin. A peak at 278 nm refers to the absorption characteristic of aromatic residues (Trp, Tyr, and Phe), while a peak at 222 nm belongs to peptide and carboxylic acid moieties of the backbone structure [27,28]. In the presence of cloxyquin, a backbone peak at 222 nm was enhanced and slightly shifted to the right at 224 nm, indicating that cloxyquin forms complex with BSA and later disturbs amide bonds of BSA structure. In contrast, cloxyquin exerted no significant changes of absorption peak around 278 nm, implying that the microenvironment around the aromatic residues is not fully exposed to the cloxyquin. In addition, pure cloxyquin exhibited absorption peaks at about 207 and 244 nm. However, its interference on BSA spectra could be subtracted out as clearly evidenced by the second order derivative spectra, which shows identical bands at around 207 and 244 nm among the spectra of BSA and BSA–cloxyquin subtracted by cloxyquin.

### 2.3. Binding Parameters of BSA–Cloxyquin Complex

To investigate deeply into the complex formation, the binding constant ($K_{b}$) and the number of binding sites (n) for BSA–cloxyquin complex were determined by Equation (3).

(3) $$\log\left(\frac{F_{0}-F}{F}\right)=\log K_{b}+n\log\left[Q\right]$$

where $F_{0}$ and F are the steady-state fluorescence intensities of BSA in the absence and presence of cloxyquin at [*Q*] concentrations, respectively.

**Figure 6:**
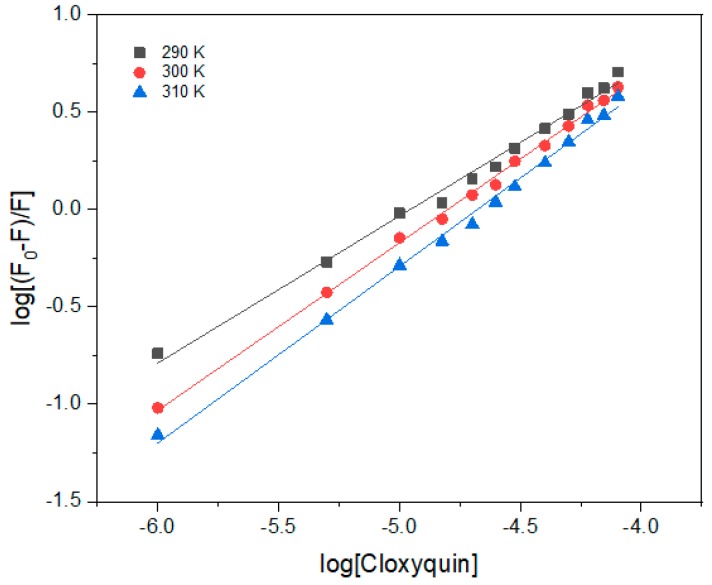
The plot of log[$\left(F_{0}\;-\;F\right)/F$] versus log[Q] at 290, 300, and 310 K. [Cloxyquin] = 0–80 µM; [BSA] = 4 µM; λ_ex_ = 280 nm; λ_em_ = 340 nm.

As presented in Figure 6 and concluded in Table 3, the $K_{b}$ values were found at the level of 10^4^ M^−1^, which were equivalent to the $K_{a}$ values obtained from the modified Stern–Volmer model (Table 2). The n values were in a range of 0.7–0.9, suggesting that cloxyquin binds to BSA with moderate affinity at about 1:1 stoichiometry. In addition, increasing the temperature of interaction resulted in the rising of $K_{b}$ and n values, demonstrating the association of hydrophobic interactions in the cloxyquin-BSA binding process. Because the temperature tends to enhance the strength of hydrophobic interactions in aqueous medium and then enlarges the association constant [29]. In contrary, if the binding process is mainly geared by electrostatic interactions and/or hydrogen bonding, decreasing $K_{b}$ is expected to be observed because the temperature tends to disfavor the binding.

To further clarify the nature of binding forces between cloxyquin and BSA, entropy (ΔS°), enthalpy (ΔH°), and Gibbs free energy (ΔG°), which are the thermodynamic parameters, were determined by Equations (4) and (5).

(4) $$\ln K_{b}=-\frac{\Delta H{}^{\circ}}{RT}+\frac{\Delta S{}^{\circ}}{R}$$

(5) ΔG°=ΔH°−TΔS°

where $K_{b}$ is the binding constant at corresponding temperature of *T* (K), which can be substituted by $K_{a}$ of the modified Stern–Volmer equation. *R* is the gas constant (8.314 J mol^−1^ K^−1^). 

The ΔG° value determines the spontaneity of reaction. Its negative value refers that the reaction is spontaneous while the positive value refers to non-spontaneous reaction. The values of ΔH° and ΔS° help elucidate the major binding force of the protein-ligand, which include hydrogen bonds (ΔH° < 0 and ΔS° < 0), van der Waals forces (ΔH° < 0 and ΔS° < 0), electrostatic/ionic interactions (ΔH° < 0 and ΔS° > 0), and hydrophobic interactions (ΔH° > 0 and ΔS° > 0) [30,31,32]. Herein, the thermodynamic parameters of BSA–cloxyquin interaction are demonstrated in Table 2 and Table 3. Both $K_{a}$ and $K_{b}$ gave the same assumption for the BSA–cloxyquin complexation. The ΔG° values were in negative quantities for all three different temperatures, indicating that cloxyquin spontaneously binds to BSA. The BSA–cloxyquin complex is more likely to occur at lower cloxyquin concentrations as indicated by its ~2.5 times lesser binding energy than those of higher concentrations. Additionally, the hydrophobic forces mainly drive the interaction of cloxyquin and BSA owing to the positive values of both ΔH° and ΔS° [33]. Its positive enthalpy change (ΔH°) also indicates the endothermic process of reaction [34].

### 2.4. Fluorescence Resonance Energy Transfer (FRET) Investigation

In some circumstances, ligand causes fluorescence quenching of its target protein via the fluorescence resonance energy transfer (FRET) process, in which the fluorophores of protein transfer its energy to the adjacent ligands (called as quenchers) by a non-radiative fashion via long-range dipole-dipole interactions. The FRET phenomenon may be considered if certain criteria are met. At first, the emission spectrum of donor fluorophore (Trp and/or Tyr of BSA) must be overlapped with the absorption spectrum of quencher (cloxyquin). Next, donor and quencher have to be situated in a close proximity to one another within 2–8 nm. Lastly, the orientations of transition dipole of the acceptor and donor must be aligned to each other [35]. FRET is an advantageous method for measuring the distance between quencher and fluorophore, which helps distinguish whether ligand binds to protein in the close proximity with Trp residues. The intensities of BSA fluorescence with and without cloxyquin were applied for calculating the fluorescence energy transfer efficiency (*E*), which was further used for estimation of the distance between cloxyquin and Trp according to the Equation (6).

(6) $$E=\frac{F_{0}-F}{F_{0}}=\frac{R_{0}^{6}}{R_{6}^{6}+r^{6}}$$

where $F_{0}$ and F are the fluorescence intensities of donor in the absence and presence of quencher, respectively. $R_{0}$ is the Förster distance at which the fluorescence probability is equal to the energy transfer probability and can be expressed as Equation (7), and *r* is the actual distance between donor and acceptor.

(7) $$R_{0}=0.211{\left[\frac{k^{2}\phi_{D}J}{n^{4}}\right]}^{\frac{1}{6}}\;$$

where $k^{2}$ is the orientation factor between donor and acceptor, $\phi_{D}$ is the fluorescence quantum yield of the donor (without acceptor), n is the refractive index of the medium, and *J* is the spectral overlap integral of the donor emission spectrum and the acceptor absorption spectrum as given by Equation (8) [34].

(8) $$J=\frac{\int_{0}^{\infty }F\left(\lambda \right)\varepsilon \left(\lambda \right)\lambda^{4}d\lambda }{\int_{0}^{\infty }F\left(\lambda \right)d\lambda }$$

where F(λ) and ε(λ) are the fluorescence intensity of the donor and the molar absorption coefficient of the acceptor at a wavelength *λ*, respectively. 

**Figure 7:**
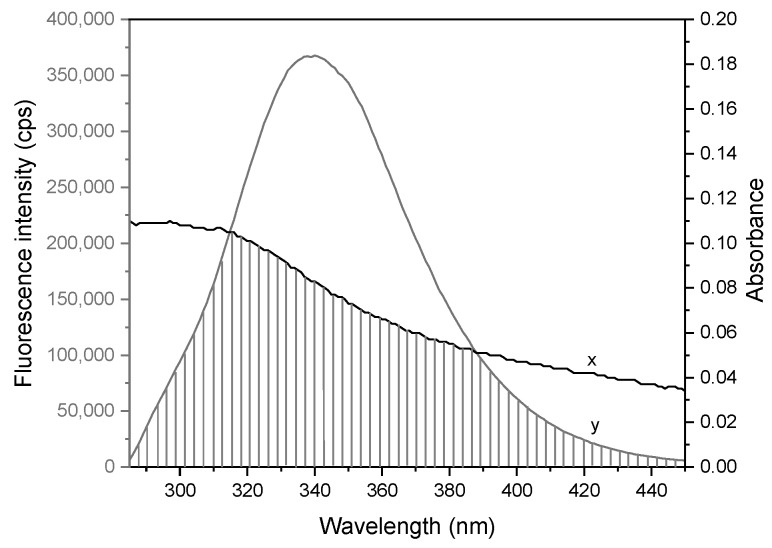
The emission and absorption spectra of cloxyquin (x) and BSA (y), respectively, at 310 K and pH 7.4 (λ_ex_ = 280 nm). A shaded region represents the overlapping area of both spectra. [BSA] = [Cloxyquin] = 4 µM.

The overlap of the BSA fluorescence emission and cloxyquin absorption spectra is represented in Figure 7. The distance (*r*) between cloxyquin and Trp was calculated based on Equations (6)–(8) with the assumption parameters of BSA-ligand interaction that *n* and $\phi_{D}$ are equal to 1.336 and 0.15, respectively [22,36]. The $k^{2}$ factor can range from 0 to 4 depending on the relative orientation of donor and acceptor. Nevertheless, there is a challenge for estimation of absolute $k^{2}$ in the real system. Practically, $k^{2}$ is assumed to be 2/3 for donor and acceptor that can tumble the orientations freely. Alternatively, the $k^{2}$ value of 0.476 can be decided for static donor-acceptor orientations. Herein, Trp residues and cloxyqiun bound in the pocket of BSA may not be able to freely randomize the orientations, so segmental motions of the structures are assumed [24]. Therefore, $k^{2}$ values of 0.476 and 0.667 were applied for calculations of cloxyquin–Trp distances. The energy transfer efficiency (*E*) was found to be 0.19 and the overlap integral (*J*) was 2.19 × 10^14^ M^−1^ cm^−1^ nm^4^, giving $R_{0}$ are equal to 2.72 and 2.88 nm for $k^{2}$ values of 0.476 and 0.667, respectively. The cloxyquin–Trp distances (*r*) are calculated to be 3.46 and 3.66 nm for static and dynamic orientations, respectively. The estimated values of $R_{0}$ and *r* fall in the range of 2–8 nm and 0.5$R_{0}$ < *r* < 1.5$R_{0}$, indicating that cloxyquin binds to the BSA in the close proximity with Trp and quenches the BSA fluorescence by radiative energy transfer process. There are two tryptophan residues of BSA, namely Trp134 and Trp213. The FRET analysis herein cannot specify exactly which tryptophan transfers its excited energy to cloxyquin because the estimation was based on the fluorescence quenching signal of both residues. However, the molecular docking models revealed that the distances between the quinoline ring of cloxyquin and the indole ring of tryptophan are in a range of 1.1 to 4.7 nm depending on the docked position (Table S1), which embrace to and well conform with the FRET analysis. 

### 2.5. Synchronous Fluorescence Analysis

**Figure 8:**
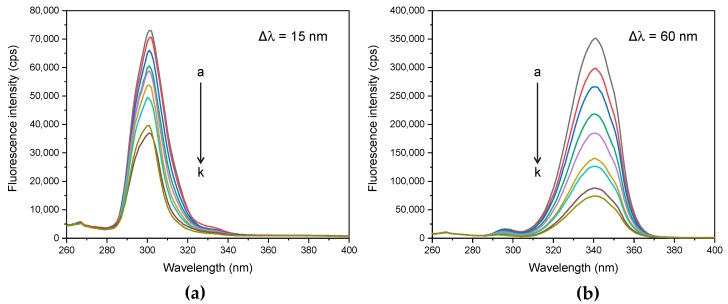
The synchronous fluorescence spectra of BSA (4 µM) in the existence of cloxyquin (a–k: 0, 1, 5, 10, 20, 30, 40, 50, 60, 70, and 80 µM) at pH 7.4 and 310 K. (**a**) Tyrosine spectra (Δλ = 15 nm) and (**b**) tryptophan spectra (Δλ = 60 nm).

To explore more information about microenvironment surrounding the BSA fluorophores, the synchronous fluorescence analysis was performed to detect the spectra of BSA–cloxyquin complex. The Tyr and Trp spectra were achieved by concurrently scanning the wavelengths of excitation and emission at constant offsets (Δλ) of 15 and 60 nm, respectively. The shift of a maximum emission peak shows the polarity changes around the residues of Tyr and Trp. A red-shift refers to the increase of polarity, while a blue-shift means to the increase of hydrophobicity at local environment of the fluorophore [22,37]. In the absence of cloxyquin, the maximum peaks of Tyr and Trp spectra were 301 and 341 nm, respectively (Figure 8). Cloxyquin dramatically reduced the fluorescence of Tyr and Trp at a concentration dependent manner. However, it exerted a quenching effect on Trp higher than Tyr. No significant peak shift was observed in both of Tyr and Trp spectra, indicating that the binding of cloxyquin to BSA does not significantly change the polarity at local environment of Tyr and Trp [38]. 

### 2.6. Investigation of Conformational Changes

#### 2.6.1. Three-Dimensional (3D) Spectrum Investigation and Dynamic Light Scattering (DLS) Analysis

**Figure 9:**
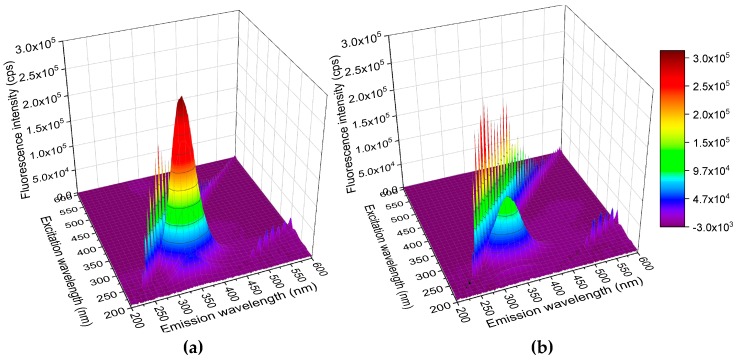
3D spectra of BSA in (**a**) the absence and (**b**) the presence of cloxyquin at 310 K, pH 7.4. [BSA] = [cloxyquin] = 4 µM.

The qualitative information of protein configuration and conformation can be explored by the measurement of three-dimensional (3D) spectra. In the absence of cloxyquin, a typical 3D spectrum of BSA was observed (Figure 9a). The spectral characteristics of Trp and Tyr were prominent at 280 nm excitation and 342 nm emission wavelengths (280/342 nm). A minor peak related to the polypeptide backbone spectrum was found at 230/342 nm. The Rayleigh scattering peaks were shown at each equivalent wavelength of excitation and emission. Addition of cloxyquin resulted in 2–3 times reduction of both major and minor peaks of BSA (Figure 9b), suggesting that cloxyquin induces the microenvironmental and conformational changes of BSA. 

**Figure 10:**
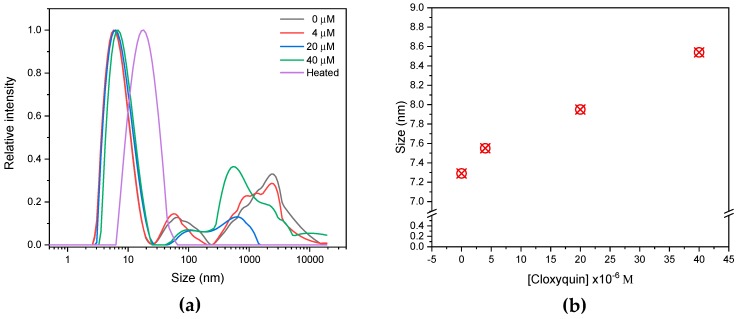
Dynamic light scattering (DLS) determination of size distribution (**a**) and hydrodynamic diameter of monomeric structure (**b**) of BSA (4 µM) upon exposure to cloxyquin (0, 4, 20, and 40 µM) for 10 min at 310 K, pH 7.4.

Besides, cloxyquin seems to promote BSA aggregation as indicated by the magnification of scattering peaks [22], so the dynamic light scattering (DLS) analysis was employed for elucidation of the aggregation events. In the absence of cloxyquin, monomeric structure of BSA was clearly evidenced at 7.29 nm diameter (Figure 10) [39,40]. The high molecular weight aggregate (HMWA) contents of BSA were also observed in a size range between 200–10,000 nm with two prominent distributions at approximately 65 and 2325 nm. In equilibration with cloxyquin, the monomeric sizes of BSA were slightly increased to 7.55, 7.95, and 8.54 nm at the BSA to cloxyquin ratios of 1:1, 1:5, and 1:10, respectively. Cloxyquin also distorted the HMWA contents to majorly populate at about 560 nm diameter. In contrast, thermally denatured BSA showed clearly monomodal size distribution at 19.39 nm without HMWA formation. Therefore, these observations indicate that the binding of cloxyquin may perturb the BSA structure, which later enhances the hydrated shell thickness and enlarges the hydrodynamic diameter of monomeric BSA. There is no clear evidence of cloxyquin-enhanced BSA aggregation; however, cloxyquin tends to reduce the size of HWMA instead. To the best of our knowledge, no report about plasma concentration of cloxyquin is available. However, in analogy to clioquinol (8-hydroxyquinoline derivative), its plasma concentration may be in a range of 13–25 µM [41] while the human serum albumin concentration is about 450–750 µM, so the ratio of serum albumin to 8-hydroxyquinoline derivative is 1:0.03 approximately. Therefore, anomalous aggregation effect of cloxyquin toward serum albumin may be negligible due to the trivial ratio of drug to serum albumin in blood circulation.

#### 2.6.2. Secondary Structure Examination by Circular Dichroism Spectroscopy

**Figure 11:**
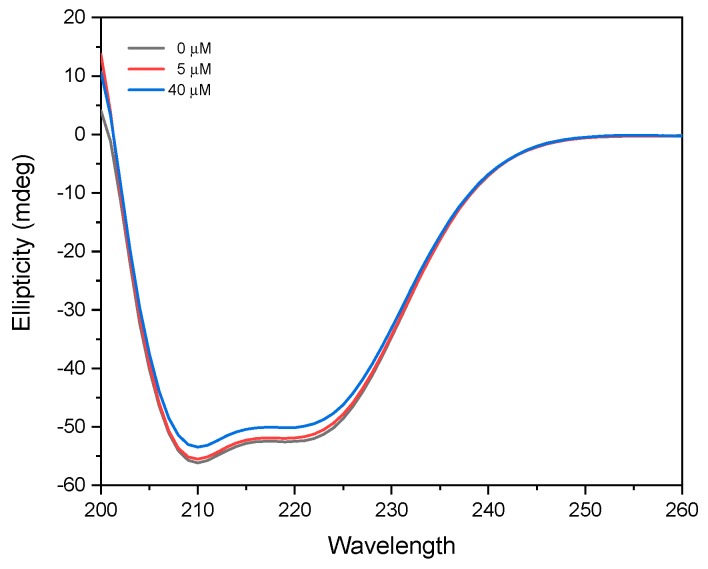
Circular dichroism (CD) spectra of 4 µM BSA in the absence (black line) and presence of 5 µM (red line) and 40 µM (blue line) cloxyquin. The measurements were performed at 310 K and pH 7.4 under a nitrogen atmosphere.

To further investigate the possibility of cloxyquin that induces the changes of BSA secondary structures, the circular dichroism (CD) spectra of BSA with and without cloxyquin were determined. A range of 200 to 260 nm was used in the CD spectrum analysis in order to display the α-helical peaks (Figure 11) and further calculate the α-helix contents at 208 nm using the following equations (Equations (9) and (10)).

(9) $$\alpha -helix\;\left(\%\right)=\;\frac{\left[-MRE_{208}-4000\right]}{\left[33,000-4000\right]}\times 100$$

where *MRE*_208_ denotes for the mean residue ellipticity (MRE) by applying Equation (10) on the observed ellipticity ($\theta_{obs}$) in millidegrees of circular dichroism data at 208 nm. A number of 33,000 is the MRE of pure α-helix and 4000 is the MRE of β-form and random coil at 208 nm.

(10) $$MRE=\frac{\theta_{obs}}{C_{p}nl\;\times 10}$$

Equation (10), $C_{p}$ is the molar concentration of the protein, n is the number of amino acid residues, and l is the path length in centimeters [42,43].

The observed CD spectra exhibited two distinct negative bands at 208 and 222 nm, representing the property of α-helix structure of BSA contributing to π-π* transition of the peptide bonds [44]. Without cloxyquin, the α-helix content of 4 µM BSA was 72.86%. Addition of 5 µM cloxyquin revealed no significant changes of BSA’s secondary structure, and its α-helix content remained to be 72.18%. Upon exposure to a high concentration of cloxyquin at 40 µM, α-helix content of BSA was reduced to be 68.44%. This observation suggested that the binding of cloxyquin might destroy the hydrogen bond networks of BSA and further provoke structural alteration and enlarge monomeric size of BSA as aforementioned in the DLS study.

### 2.7. Probing the Binding Sites with Site Marker Displacement Studies

Serum albumin consists of various binding pockets for small molecules. Site I and site II are predominantly reported as potential binding sites for aromatic and heterocyclic compounds, situated in hydrophobic pockets on subdomains IIA and IIIA, respectively. Recently, site III situated in the hydrophilic pocket on IB has been recognized as the major binding site for various compounds [18]. To determine the favorable binding sites of cloxyquin on BSA, competitive displacement studies with the well-known site markers were employed using warfarin, ibuprofen, and digitoxin (Figure 1) as a site probe for site I, site II, and site III, respectively. Table 4 shows the BSA–cloxyquin binding constants in the presence of different site probe markers. Cloxyquin seems to share its binding site with ibuprofen and digitoxin as indicated by the slight reduction of binding constants about 18–29%. This could be due to the aromatic ring of ibuprofen/pyran ring of digitoxin, which might mimic the binding at the pocket site of BSA. In other words, ibuprofen and digitoxin may induce negative cooperative binding toward cloxyquin-BSA. In contrary, warfarin resulted in 41% enhancement of the binding constant, suggesting the allosteric effect of warfarin toward cloxyquin. Such allosteric effect may be due to the bulky/steric size of phenylcoumarin ring of the warfarin.

### 2.8. Molecular Docking for Elucidating the Major Binding Sites of Cloxyquin on BSA

**Figure 12:**
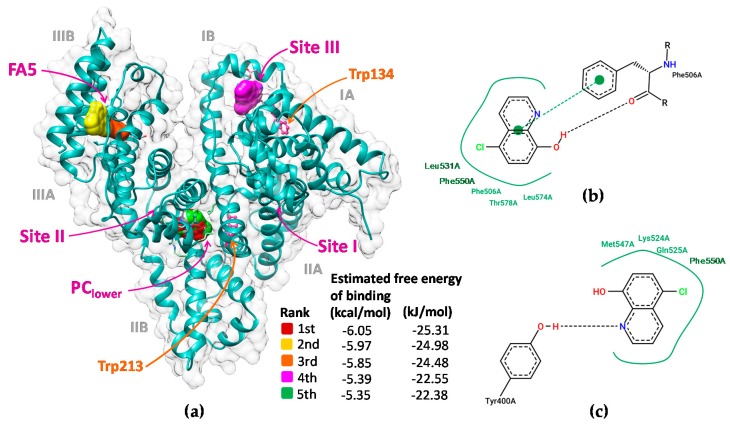
(**a**) The ribbon view and surface topology demonstrating the molecular interactions of cloxyquin in different proposed binding sites on BSA structure. Trp134 and Trp213 are shown in pink ball and stick structures for visual guidance. (**b**,**c**) show two-dimensional diagram showing interaction networks between cloxyquin and BSA at the 2nd and 3rd docking poses, respectively.

The molecular docking simulations produced 20 docking poses, mainly located at subdomains IB and IIIB and a groove between subdomains IIA and IIIA (Figure S2). The top 5 energy ranked docking poses of cloxyquin were situated on BSA at 3 different regions (Figure 12a), including lower area of the protein cleft between subdomains IIA and IIIA (PC_lower_), fatty acid binding site 5 (FA5), and fatty acid binding site 3 (FA3) or site III. Their estimated binding energies were in well agreement with the binding energies obtained from quenching investigation (Figure 12a, Table 2 and Table 3). By analysis of all top 5 docking poses, the quinoline ring of cloxyquin is distant from the indole ring of tryptophan residues in a range between 1.1 and 4.7 nm (Table S1), which embrace to and coincide with the FRET analysis. The cloxyquin–Trp distances of the 2nd and 3rd rank docking poses seem to fit best with the FRET distances. The 2nd and 3rd poses were located in the same pocket at FA5 with different orientations. Cloxyquin is far from Trp134 and Trp213 in a range of 3.8–4.7 nm, nearly matched with the estimated FRET distance (3.46–3.66 nm). The interaction mainly occurs by the hydrophobic forces among the quinoline ring of cloxyquin and the Phe550, Leu531, and Lue574 residues of BSA (Figure 12b,c). Phe506 and Tyr400 also exerted the π-π interaction and the hydrogen bonding with the cloxyquin ring. Both 2nd and 3rd docking poses equally showed the binding force at about −24 kJ/mol. All of these findings are well agreement with the thermodynamic estimation and the FRET analysis. Besides, the 1st and 5th rank docking poses consigned the cloxyquin in the same region at PC_lower_ nearby the site II region. This information may speculate why the binding constant of BSA–cloxyquin was reduced by ibuprofen as observed in a competitive displacement study (Table 4). The binding of ibuprofen into site II may cause conformational changes of the PC_lower_ region and subsequently hinders the binding of cloxyquin. Hydrophobic interactions play a key role in the cloxyquin binding at PC_lower_ pocket, which is consistent with a thermodynamic interpretation. A quinoline scaffold of cloxyquin structure exerted hydrophobic interaction with Leu197 and Leu480 residues (Figure S3). The hydrogen bond formations between a hydroxyl group of cloxyquin with Arg483 and Ser453 were also observed. Nevertheless, the PC_lower_ pocket is very adjacent to Trp213 at about 1.2 nm, arising a paradoxical question why the binding of cloxquin did not provoke much FRET phenomenon. So, its possibility to be a favorable binding site of cloxyquin is weaken. Furthermore, in accordance with the competitive displacement results, the binding of cloxyquin onto site III of BSA was evidenced in the 4th docking pose. Cloxyquin majorly forms the hydrogen bonding with Glu125, which, however, is unlikely aligned with other discoveries. Therefore, the integration of multi-spectroscopic, thermodynamic, FRET, and docking analysis seem to indicate that the FA5 pocket of serum albumin is the most favorable site for binding with cloxyquin.

## 3. Materials and Methods

### 3.1. Chemicals and Reagents

Cloxyquin, warfarin, ibuprofen, digitoxin, and bovine serum albumin (BSA; >98% purity with essentially fatty acid free) were received from Sigma-Aldrich (St. Luis, MO, USA), and other chemicals were analytical grade. Cloxyquin, warfarin, ibuprofen, and digitoxin were diluted in absolute methanol and kept away from light until use. BSA stock solution was prepared in Tris-HCl buffer containing 10 mM Tris-HCl and 150 mM NaCl at pH 7.4. The stock concentration was quantitated by spectrophotometric method using a molar extinction coefficient of 43,824 M^−1^cm^−1^ at 280 nm wavelength. The BSA stock solution was aliquoted and stored at −20 °C, which afterward was freshly diluted for each experiment. All studies were accomplished in aqueous environment of Tris-HCl buffer if were not otherwise stated. 

### 3.2. Steady-State Fluorescence Measurement

BSA (4 µM) was titrated with various concentrations of cloxyquin (0, 1, 5, 10, 15, 20, 25, 30, 40, 60, 70, and 80 µM) at different temperatures including 290, 300, and 310 K. The reactions were carried out in duplicate and its fluorescence spectra were detected in a range of 285–450 nm upon the excitation wavelength of 280 nm by using a QuantaMaster™ 40 spectrofluorometer (Photon Technology International, Inc., London, ON, Canada). The bandwidths of both excitation and emission units were set to be 1 nm. The inner filter effect of fluorescence signals was eradicated by the Equation (11):

(11) $$F_{cor}=F_{obs}\times e^{\frac{\left(A^{ex}-A^{em}\right)}{2}}$$

where *F_cor_* and *F_obs_* are the corrected and measured fluorescence intensities, respectively. $A^{ex}$ and $A^{em}$ are the absorbances of cloxyquin reading at excitation and emission wavelengths of related fluorescence measurement, respectively [45].

### 3.3. Absorption Measurement

The UV-Vis spectra of BSA (4 µM) with (1–80 µM) and without cloxyquin were determined by a NanoDrop™ Micro-UV/Vis spectrophotometer (Thermo Fisher Scientific, Inc., Bartlesville, OK, USA). The detection was carried out using 1 cm path length quartz cuvette with a scanning interval of 0.5 nm at 310 K temperature. To explore more intrinsic information, the measured absorption spectra were subjected to the second order derivative transformation with a Savitzky–Golay smoothing function at 20 data points.

### 3.4. Site Marker Competitive Analysis

Warfarin, ibuprofen, and digitoxin (Figure 1) were selected as the well-known site markers for probing the possibility of cloxyquin binding into site I, site II, and site III of BSA, respectively. An equal molar concentration (4 µM) of BSA and each site marker was incubated with different concentrations of cloxyquin ranging from 0–80 µM at physiological temperature (310 K). After 10 min of incubation, the fluorescence spectrum of each solution was recorded as the aforementioned setup.

### 3.5. Dynamic Light Scattering (DLS) experiments

The DLS analysis was employed to investigate the aggregation of BSA as a result of cloxyquin exposure by using a Litesizer^TM^ 500 (Anton Paar GmbH, Graz, Austria). BSA (4 µM) was incubated with various concentrations of cloxyquin (0, 4, 20, and 40 µM) for 10 min at 310 K, resulting in the BSA:cloxyquin molar ratios of 1:0, 1:1, 1:5, and 1:10, respectively. All solutions were filtrated through 0.22 µm syringe filter (JET BIOFIL®, Guangzhou, China) prior subjected to DLS experiments. The particle sizes were estimated on the basis of autocorrelation function at a scattering angle of 90° and a maximum number of measurements of 60 times under 1 mL sample volume in a temperature-controlled quartz cell. In addition, BSA (4 µM) was heated at 80 °C followed by DLS measurement as a denatured BSA control.

### 3.6. Circular Dichroism (CD) Spectrometry

A Jasco J-815 spectropolarimeter (JASCO International Co. Ltd., Tokyo, Japan) was used to determined CD spectra of BSA (4 µM) with (5 and 40 µM) and without cloxyquin using a 1 mm path length cuvette at 310 K under constant nitrogen flush. The CD spectra were documented ranging from 200–260 nm with an interval of 1 nm and a scan rate of 60 nm/min.

### 3.7. Molecular Docking

The high-resolution crystal structure (2.47 Å) of BSA was received from the Protein Databank (PDB) with an identification number of 4A5S. The structure is available as a homodimer of chains A and B in complexation with polyethylene glycol (PEG). For molecular docking study, only chain A was selected and then removed all water molecules and heteroatoms. The structure of cloxyquin was downloaded from ZINC database with an entry code of 1209 (http://zinc.docking.org). The docking experiment was accomplished by the Autodock tool as a web service of DockingServer (https://www.dockingserver.com/web) [46]. The calculation was performed in a grid maps of 90 × 90 × 90 Å (x, y, and z) by consecutively probing the cloxyquin at 0.375 Å grid point. A box center was set at 8.52, −21.57, and 106.69 of x, y, and z coordination, respectively. The resulting docking poses were analyzed and visualized by the UCSF chimera 1.13 (https://www.cgl.ucsf.edu/chimera/), the discovery studio 2016 (http://3dsbiovia.com/), and the PoseView (https://www.biosolveit.de/PoseView/).

## 4. Conclusions

To provide essential information for taking a step closer to the application of cloxyquin as a therapeutic drug, the interaction of cloxyquin and serum albumin has been investigated. Cloxyquin majorly interacts with BSA via static process by the formation of BSA–cloxyquin complex. Cloxyquin seems to favorably bind into the fatty acid binding pocket 5 (FA5) of BSA structure. Its quinoline scaffold exerts hydrophobic interaction with Phe550, Leu531, and Leu574 residues together with the formation of π–π interaction and hydrogen bonding with Phe506 and Tyr400, respectively. Cloxyquin interacts with BSA as a biphasic behavior with a moderate binding constant about 10^4^ M^−1^, indicating a critical role of serum albumin as a carrier of cloxyquin in blood stream. In addition, cloxyquin can cause slight enlargement of monomeric BSA, but its anomalous effect on BSA aggregation may be insignificant because of its tiny molar ratio toward BSA in blood circulation. 

## Figures and Tables

**Table 1:** Stern–Volmer constants for BSA–cloxyquin interaction at difference temperatures.

T (K)	$K_{sv}\;\times \;10^{4}\;(M^{-1})$	$k_{q}\;\times \;10^{12}\;(M^{-1}\;S^{-1})$	*r* ^2^
290	6.373 ± 0.12	6.373 ± 0.12	0.9978
300	5.415 ± 0.08	5.415 ± 0.08	0.9988
310	4.566 ± 0.06	4.566 ± 0.06	0.9990

**Table 2:** The modified Stern–Volmer association constants for the accessible fluorophores ($K_{a}$), the fractions of accessible fluorophore ($f_{a}$), and the thermodynamic parameters derived from the $K_{a}$ values of BSA in the presence of cloxyquin.

T (K)	[Cloxyquin] ^1^	$K_{a}\;\times \;10^{4}\;(M^{-1})$	$f_{a}$	*r* ^2^	$\Delta H{}^{\circ}\;(\mathrm{kJ}\;{\mathrm{mol}}^{-1})$	$\Delta S{}^{\circ}\;(J\;{\mathrm{mol}}^{-1}K^{-1})$	$\Delta G{}^{\circ}\;(\mathrm{kJ}\;{\mathrm{mol}}^{-1})$
290	High	0.7870 ± 0.03	0.9567 ± 0.01	0.9965			−21.70 ± 0.3
	Low	3.1419 ± 0.09	0.6402 ± 0.09	0.9922			−52.73 ± 0.7
300	High	0.6353 ± 0.05	0.9615 ± 0.02	0.9939	21.6 ± 1.5	0.4 ± 0.3	−21.70 ± 0.4
	Low	1.4231 ± 0.06	0.6992 ± 0.07	0.9990	38.8 ± 0.9	48.2 ± 0.5	−53.21 ± 0.9
310	High	0.4408 ± 0.07	1.0016 ± 0.02	0.9942			−21.71 ± 0.5
	Low	1.1204 ± 0.01	0.6425 ± 0.01	0.9988			−53.69 ± 1.1

^1^ Low and high concentrations of cloxyquin are defined in a range of 1–25 µM and 15–80 µM, respectively.

**Table 3:** Binding constants, numbers of binding site, and thermodynamic parameters derived from the $K_{b}$ values of BSA–cloxyquin complex.

T (K)	$\begin{array}{c} K_{b}\;\times \;10^{4}\\ \left(M^{-1}\right) \end{array}$	*n*	*r* ^2^	$\begin{array}{c} \Delta H{}^{\circ}\\ \left(\mathrm{kJ}\;{\mathrm{mol}}^{-1}\right) \end{array}$	$\begin{array}{c} \Delta S{}^{\circ}\\ \left(J\;{\mathrm{mol}}^{-1}K^{-1}\right) \end{array}$	$\begin{array}{c} \Delta G{}^{\circ}\\ \left(\mathrm{kJ}\;{\mathrm{mol}}^{-1}\right) \end{array}$
290	0.5506 ± 0.01	0.755 ± 0.02	0.9915			−21.0 ± 0.2
300	1.3590 ± 0.01	0.861 ± 0.01	0.9975	43.4 ± 2.9	222.0 ± 10.9	−23.2 ± 0.3
310	1.7459 ± 0.01	0.907 ± 0.02	0.9954			−25.5 ± 0.4

**Table 4:** The binding constants ($K_{b}$) and number of binding sites (n) of BSA–cloxyquin complex in the presence and the absence of site markers.

Site Marker	$K_{b}\;\times \;10^{4}\;(M^{-1})$	*n*	*r* ^2^
Without	1.746 ± 0.01	0.91 ± 0.02	0.9954
Warfarin	2.454 ± 0.01	0.94 ± 0.02	0.9943
Ibuprofen	1.424 ± 0.01	0.89 ± 0.02	0.9923
Digitoxin	1.239 ± 0.01	0.88 ± 0.04	0.9828

## Supplementary Materials

Supplementary materials can be found at https://www.mdpi.com/1422-0067/21/1/249/s1.

## Abbreviations

SA	Serum Albumin
BSA	Bovine Serum Albumin
HSA	Human Serum Albumin
MIC	Minimum Inhibition Concentration
TRESK	TWIK-related spinal cord potassium channel
FA3	Fatty acid binding site 3
FA5	Fatty acid binding site 5
PC_lower_	Protein cleft between subdomains IIA and IIIA
K	Kelvin
UV-Vis	Ultraviolet and Visible light
CD	Circular Dichroism spectroscopy
FRET	Fluorescence Resonance Energy Transfer
DLS	Dynamic Light Scattering
Arg	Arginine
GluLeu	GlutamicLeucine
Phe	Phenylalanine
Ser	Serine
Trp	Tryptophan
Tyr	Tyrosine
