# Supplementary material for: Insight into the Molecular Interaction of Cloxyquin (5-chloro-8-hydroxyquinoline) with Bovine Serum Albumin: Biophysical Analysis and Computational Simulation
Source: Int J Mol Sci. 2019 Dec 30;21(1):249. doi: 10.3390/ijms21010249 (PMC6981711; doi:10.3390/ijms21010249)
Supplement: Supplementary file 1 [file ijms-21-00249-s001.pdf]

Supplement information

Insight into the Molecular Interaction of Cloxyquin (5-chloro-8-hydroxyquinoline) with Bovine Serum Albumin: Biophysical Analysis and Computational Simulation

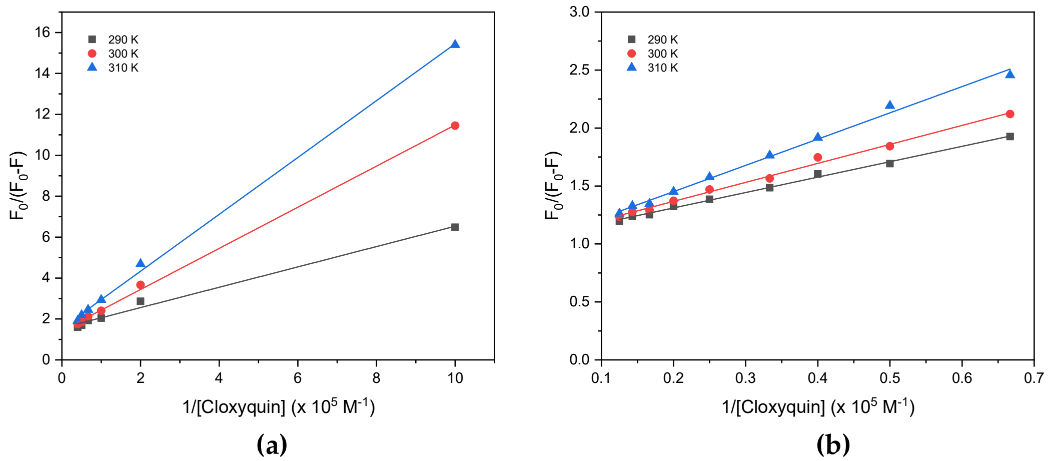

Figure S1. The modified Stern-Volmer plots of BSA after exposed with low (1–25 μM; **a**) and high (15–80 μM; **b**) concentrations of cloxyquin at 290, 300, and 310 K. [BSA] = 4 μM; λ_{ex} = 280 nm; λ_{em} = 340 nm.

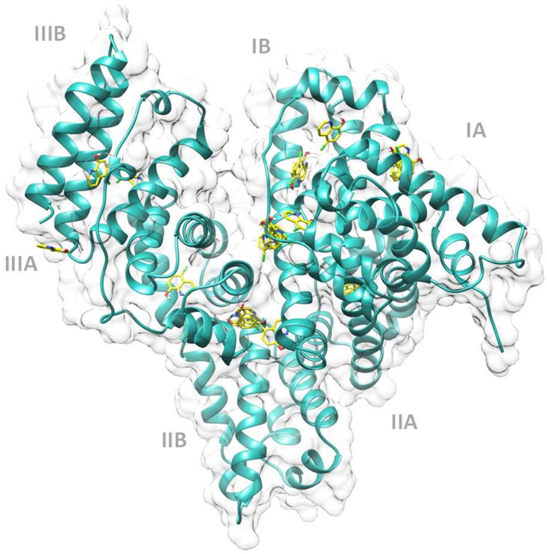

Figure S2. Superimposition of 20 docking poses (yellow color) of cloxyquin onto the ribbon and surface topology of BSA.

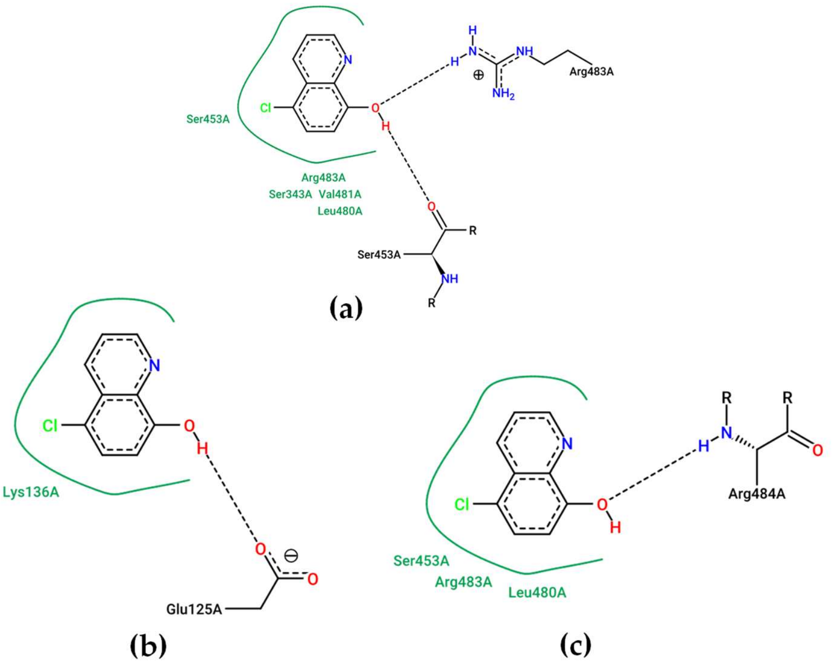

Figure S3. Two-dimensional diagrams showing interaction networks between cloxyquin and BSA of the (**a**) 1st, (**b**) 4th, and (**c**) 5th rank docking poses.

Table S1. Distance between cloxyquin and Trp residues of BSA estimated from five docked poses.

Docked poses	Estimated Distance (nm)	
	Trp134	Trp213
1st pose	3.2	1.1
2nd pose	4.7	4.3
3rd pose	3.8	4.1
4th pose	1.2	3.2
5th pose	3.4	1.2
